# Novel Development of Phosphate Treated Porous Hydroxyapatite

**DOI:** 10.3390/ma10121405

**Published:** 2017-12-08

**Authors:** Kazuya Doi, Yasuhiko Abe, Reiko Kobatake, Yohei Okazaki, Yoshifumi Oki, Yoshihito Naito, Widyasri Prananingrum, Kazuhiro Tsuga

**Affiliations:** 1 Department of Advanced Prosthodontics, Hiroshima University Graduate School of Biomedical and Health Sciences, 1-2-3, Kasumi, Minami-ku, Hiroshima 734-8553, Japan; abey@hiroshima-u.ac.jp (Y.A.); Reiko1122@hiroshima-u.ac.jp (R.K.); okazaki-yoh@hiroshima-u.ac.jp (Y.O.); yos-oki14@hiroshima-u.ac.jp (Y.O.); tsuga@hiroshima-u.ac.jp (K.T.); 2 Oral Implant Center, Tokushima University Hospital, 2-50-1, Kuramoto-machi, Tokushima 770-8503, Japan; yoshi11@tokushima-u.ac.jp; 3 Department of Dental Material Science and Technology, Hang Tuah University, Jalan Arief Rachman Hakim No. 150, Sukolilo, Surabaya 60111, Indonesia; widyasri.prananingrum@hangtuah.ac.id

**Keywords:** porous hydroxyapatite, phosphoric acid-etching, phosphate treated hydroxyapatite

## Abstract

Phosphoric acid-etching treatment to the hydroxyapatite (HA) surface can modify the solubility calcium structure. The aim of the present study was to develop phosphate treated porous HA, and the characteristic structures and stimulation abilities of bone formation were evaluated to determine its suitability as a new type of bone graft material. Although the phosphoric acid-etching treatment did not alter the three-dimensional structure, a micrometer-scale rough surface topography was created on the porous HA surface. Compared to porous HA, the porosity of phosphate treated porous HA was slightly higher and the mechanical strength was lower. Two weeks after placement of the cylindrical porous or phosphate treated porous HA in a rabbit femur, newly formed bone was detected in both groups. At the central portion of the bone defect area, substantial bone formation was detected in the phosphate treated porous HA group, with a significantly higher bone formation ratio than detected in the porous HA group. These results indicate that phosphate treated porous HA has a superior surface topography and bone formation abilities in vivo owing to the capacity for both osteoconduction and stimulation abilities of bone formation conferred by phosphoric acid etching.

## 1. Introduction

Hydroxyapatite [Ca_10_(PO_4_)_6_(OH)_2_] (HA) has been applied as an artificial bone graft material because of its excellent biocompatibility and osteoconduction ability [1]. Osteoconduction plays a crucial role in bone formation, which largely depends on the structure of a material. HA with a porous structure shows superior osteoconduction because it has an interconnected structure, which allows for the efficient migration of osteogenic cells and vascularization from surrounding bone [1,2]. Interconnected porous hydroxyapatite (IP-CHA), a type of porous HA, has a systematic arrangement of uniform pores with interconnections, and has been successfully used as a bone graft material for tissue engineering [3,4,5]. Despite the superior osteoconduction of porous HA, the ability of bone regeneration is not predictable, owing to the lack of stimulation abilities of bone formation. The solubility of calcium phosphate is strongly correlated with osteogenic differentiation [6,7]. HA is prepared by exposure to a high sintering temperature to obtain highly crystalline HA (Ca/P ratio: 1.67) [8], which shows sufficient mechanical strength. However, HA is relatively insoluble in vivo. By contrast, β-tricalcium phosphate [Ca_3_(PO_4_)_2_] (β-TCP) is another major type of bone graft material that shows good bioabsorption in vivo because of elution phosphate ions at absorption (Ca/P ratio: 1.50) [9,10]. However, β-TCP is fragile, and its poor post-operative mechanical strength is the main drawback for clinical application [11,12].

Therefore, it is necessary to develop a simple and reproducible approach to confer the porous HA surface with suitable osteoinductive abilities. Many surface modification treatments have been investigated to accelerate bone formation. Sand-blasted and acid-etched titanium increase the surface roughness, which can promote osteoblast activities [13]. However, with these surface treatments, the modified portion is limited to only a thin layer at the outer surface of porous scaffolds [14,15]. Furthermore, in most of these studies, the treatments were performed on a titanium surface, and rarely on porous HA surfaces. Thus, a novel and simple fabrication process for porous HA with a stimulation abilities of bone formation is required. To address this challenge, we previously established a phosphoric acid-etching treatment to modify the HA surface [16]. Indeed, phosphoric acid etching reduced the Ca/P ratio of the HA surface from 1.67 to 1.50, which is consistent with the ratio for β-TCP, indicating that the material was conferred with stimulation abilities of bone. This was confirmed by an in vitro study demonstrating that the phosphoric acid-treated HA surface significantly promoted the adhesion, proliferation, and differentiation of osteoblastic cells. These results indicated that phosphoric acid treatment can modify a porous HA surface to achieve a biocompatible material with suitable stimulation abilities of bone formation to serve as a bone graft material.

The aim of the present study was to develop a new, simple approach to fabricating phosphate treated porous HA scaffolds, and to evaluate the bone formation abilities of the material in vivo by histological analysis.

## 2. Results

### 2.1. Structure Characteristics Properties of Porous Hydroxyapatite (HA) and Phosphate Treated HA

**Figure 1:**
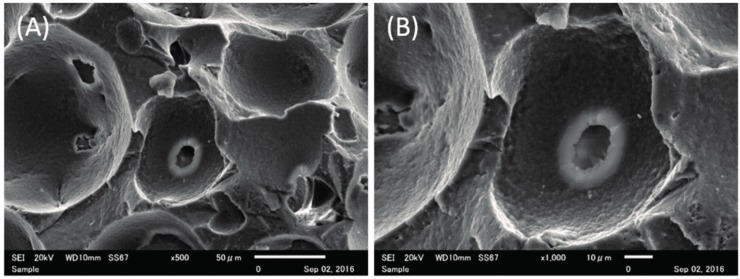

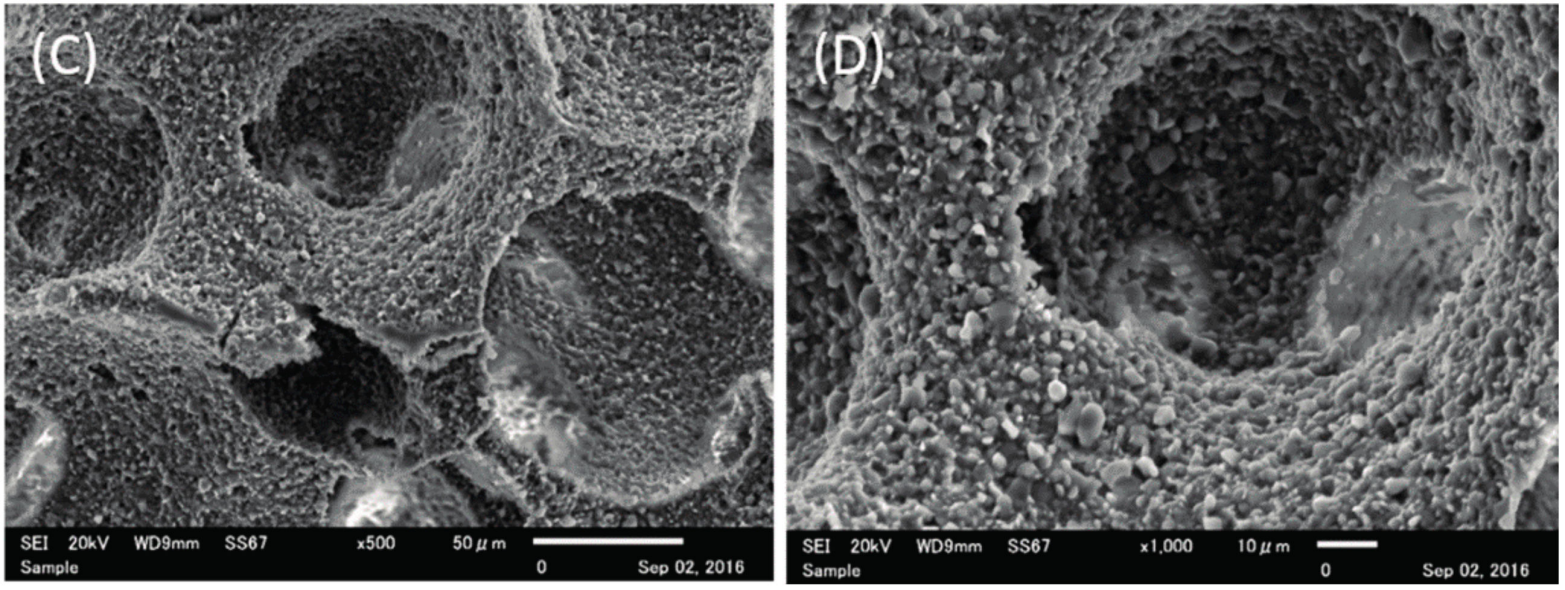
Scanning electron microscope (SEM) image of each sample. ((**A**,**B**) porous hydroxyapatite (HA), (**C**,**D**) phosphate treated HA). Low-magnification (500×) images showing the three-dimensional structure with maintenance of interporous connections (**A**,**B**). High-magnification (1000×) images showing the surface of porous HA with a smooth inner structure (**C**) and phosphate treated HA with a micrometer-scale rough structure created on the pores (**D**).

Figure 1 shows the scanning electron microscope (SEM) images of porous and phosphate treated HA; a three-dimensional structure was observed for both materials. High magnification revealed that porous HA had a smooth inner pore surface, whereas phosphate treated HA showed micrometer-scale roughness on the inner surface of the pores. As shown in Table 1, the total porosity of phosphate treated HA was almost equal that of porous HA (* *p* = 0.0412). In addition, the compressive strength of porous HA was significantly higher than that of phosphate treated HA (* *p* = 0.0003).

### 2.2. Histological Observations and Histomorphometric Analyses

**Figure 2:**
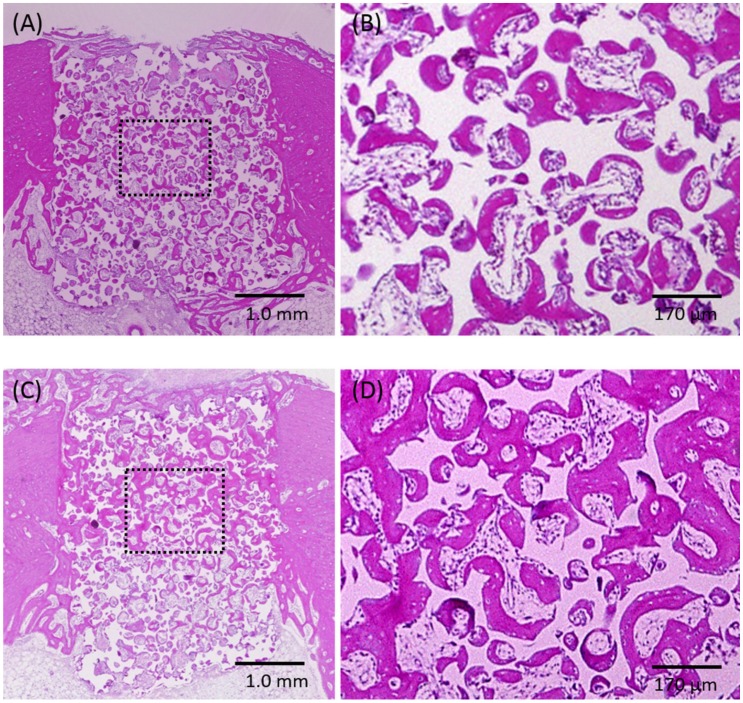
Histological specimens of porous HA (**A**,**B**) and phosphate treated HA (**C**,**D**).

Figure 2 shows representative histological images from the samples obtained from rabbits grafted with the porous HA and phosphate treated HA.

In both groups, newly formed bone ingrowth could be observed into the scaffolds from the marginal side of the parent bone over the entire defect; the defect space contained regenerated bone tissue (A, C). Magnification of the squared areas, showing the central area of the defect; the pores are filled with woven bone, lamella bone, and connective tissue. Substantially more bone tissue could be detected in the phosphate treated HA group (D) compared with the porous HA group (B).

Osteoconduction from the parent bone site was detected for both groups. Ingrowth of newly formed bone was observed in the pore walls. In the phosphate treated HA group, newly formed bone was detected, not only on the side portions, but also in the central portion of the defect. However, a similar degree of bone formation areas was observed on the whole area between the two groups, with no significant difference in bone formation. On the other hand, significantly difference was detected in the central area for the phosphate treated HA compared to that observed for the porous HA group (Table 2, * *p* = 0.0272).

## 3. Discussion

Osteoconduction and stimulation abilities of bone formation are the major factors required for effective biomaterials for use in bone grafting. Osteoconduction is defined as the ability of bone ingrowth into the material, and largely depends on the structural properties of the material, such as surface topography, porosity, and interconnectivity [17].

In general, the optimal pore size for osteoconduction is considered to be in the range of 150–500 μm [18,19], which allows for efficient migration of osteogenic cells. In the present study, we used IP-CHA to prepare phosphate treated HA with phosphoric acid-etching treatment; IP-CHA has a porosity of 75%, average pore size of 150 μm, and an average interconnected pore size of 40 μm—favorable properties for osteoconduction [3]. According to SEM observations, the phosphate treated HA has a three-dimensional porous structure similar to porous HA, and the interconnected pores remained after treatment. The porosity of phosphate treated HA was slightly higher than that of porous HA, which was in the recommended range for osteoconduction. The surface of phosphate treated HA was slightly decalcified due to the phosphoric acid-etching treatment, resulting in a micrometer-scale rough structure on the pores. Our previous in vitro study demonstrated that the phosphoric acid-treated HA surface showed favorable initial cell adhesion ability and wettability with increased hydrophilicity [16]. Therefore, this phosphate treated HA was expected to show superior osteoconduction because the porous structure is similar to that of porous HA, but with a more preferable surface topography for cell attachment. Moreover, this treatment could confer the porous HA surface with solubility and a modified surface topography. However, one disadvantage of this method is the reduction in mechanical strength due to decalcifying Ca from porous HA scaffolds. In fact, the compressive strength of the phosphate treated HA-based scaffold decreased by about one third compared with that of the porous HA-based scaffold. In general, the mechanical strength of human sponge bone ranges from 0.2 to 4 MPa [20]. Tamai et al. [3] reported the initial compressive strength of porous HA: IP-CHA is around 8–10 MPa, which increased steadily in parallel with bone ingrowth into the pores. In the animal study, the strength of implanted porous HA after 12 weeks was 3-times higher than that of the initial strength. Therefore, the mechanical strength of phosphate treated HA would be expected to increase after implantation due to ingrowth bone formation.

In line with this prediction, histological observations showed the ingrowth of newly formed bone into the pores for both the phosphate treated HA and porous HA groups. A similar amount of newly formed bone from the parent bone site was detected along the periphery in the pores of both the phosphate treated HA and porous HA groups, with no significant difference in the bone formation area over the whole defect region. This indicates that both phosphate treated HA and porous HA have good osteoconductive ability because of the three-dimensional porous structure of the material. However, the bone formation area at the central area of phosphate treated HA was higher than that of porous HA. This finding likely reflects a difference in the stimulation abilities of the two materials. Stimulation abilities of bone formation is affected as the ability for osteogenic differentiation. HA does not have intrinsic osteoinductive ability in the absence of additional growth factors such as bone morphogenetic protein. Several reports have demonstrated that the absorption of phosphate can affect osteogenic differentiation, and it is considered that increasing the calcium phosphate concentration can affect the osteoblast activity or adsorption of osteogenic-related proteins [8,21]. Harris and Cooper [22] compared the effects of HA, coral HA, bovine bone, and an HA/β-TCP composite that were respectively loaded with mesenchymal stem cells and grafted on the backs of severe combined immunodeficiency mice. Only the HA/β-TCP composite graft induced osteoinduction, due to its ability to dissolve phosphoric acid. In particular, TCP in the HA/β-TCP composite is a soluble CaPO_4_ mineral [8]. As mentioned above, phosphoric acid treatment modified the solubility calcium structure of the HA surface, which is essentially the elution of phosphoric acid and calcium ions from HA [23]. Our previous in vitro study showed that after seeding osteoblastic cells on a phosphoric acid-etched HA disk, cell proliferation and alkaline phosphatase activity were increased relative to those seeded on a non-treated HA disk [16]. Upon placement, migrated osteogenic cells might adhere to the pore walls, followed by proliferation and differentiation, leading to the formation of new bone tissue. These results of the responses of osteoblastic cells suggested that treatment of the HA surface with phosphoric acid etching would be an effective method for fabricating a graft material to enhance bone formation, which is attributed to the modified solubility calcium structure on the surface.

In the present study, the surface structure of hydroxyapatite was modified by phosphoric acid treatment. Our previous study reported about the simple phosphoric acid treatment method to modify the bioactive surface of porous sintered HA polymorphs as porous HA [16]. The flat HA block used in the previous study was fabricated with similar chemical composition to porous HA. Therefore, the same surface modification the flat HA block treated with phosphoric acid would be established the porous HA structure.

In particular, the key finding of the increased bone formation in the central portion of the defect suggests that phosphate treated HA bioactive HA has both osteoconductive and osteoinductive ability in vivo, representing a promising material for tissue engineering.

Both the present study and our previous in vitro study demonstrated that phosphate treated HA bioactive HA has a surface topography superior to that of porous HA, including improved hydrophilicity, with a micrometer-scale rough surface to allow for elution of phosphoric acid. Accordingly, phosphate treated HA resulted in superior bone formation in vivo using a rabbit model. The results of this study should help to guide the development of new bone graft materials for various applications.

## 4. Methods

### 4.1. Fabrication of Phosphoric Acid-Treated Porous HA 

**Figure 3:**
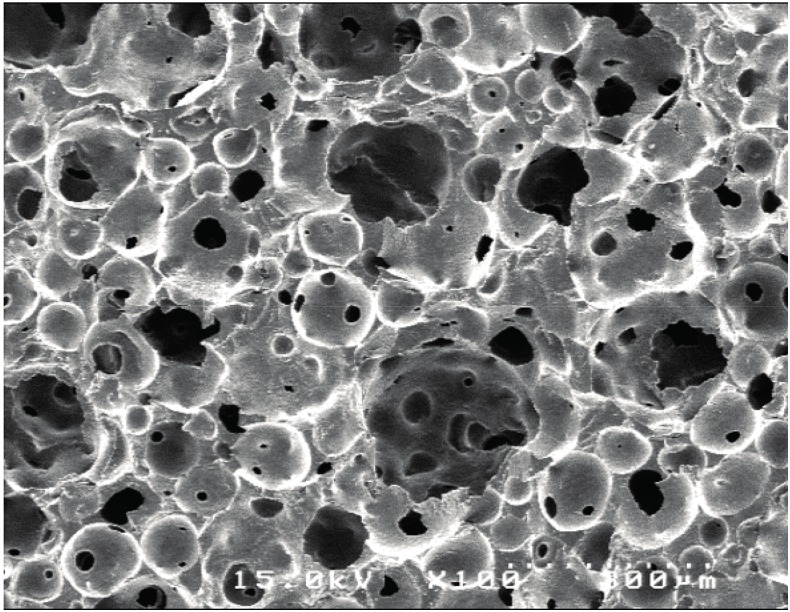
SEM image of the interconnected porous hydroxyapatite (IP-CHA) scaffold. Almost all pores are interconnected.

Two cylinder-type IP-CHAs (large: φ3.7 mm, height 7.0 mm; small: φ3.0 mm, height 3.0 mm; NEO BONE^®^, Covalent Materials, Tokyo, Japan) were prepared as scaffolds. Each type of scaffold had 75% porosity with a mean pore diameter of 150 µm, and each pore was connected via 40-µm-diameter interconnecting pore channels (Figure 3).

These IP-CHA scaffolds were soaked in a 30% phosphoric acid [H_3_PO_4_] (lot No. T1949; Sigma-Aldrich Japan, Tokyo, Japan) solution for 1 min at 25 °C under centrifuged (1000 rpm, 1 min). Then, the solution was aspirated, followed by rinsing three times with distilled water. The obtained samples were dehydrated and then sterilized via pressure-steaming. Ultimately, phosphate treated HA was prepared, and the IP-CHA was used as the porous HA material (control).

### 4.2. Structure Characterization

#### 4.2.1. Scanning Electron Microscope (SEM) Observation

Before observation, each small-type sample was sputtered with Pt-Pd to make the surface conductive, and then set on the sample stage with carbonate adhesive tape. The porous structure of each sample was observed by SEM (JMS–7300, Nihon Denshi Oyo Co. Ltd., Tokyo, Japan) at 500× and 1000× magnifications.

#### 4.2.2. Porosity Measurements

The total porosity of each small-type sample (*n* = 6) was measured on the basis of the apparent volume calculated from the specimen dimensions and the actual volume evaluated using a helium pycnometer (Accupyc II 1340, Micromeritics Instrument Corp., Norcross, GA, USA), according to the method reported by Semel and Lados [24].

#### 4.2.3. Measurement of Mechanical Strength

Compression tests were performed using an Autograph system (Autograph AGS-X 5kN, Shimadzu Seisakusho, Kyoto, Japan). The compression speed was set to 0.1 mm/min. Testing was performed vertically toward each large-type sample (*n* = 4). Load was applied to the sample until it fractured.

### 4.3. Evaluation of Bone Formation In Vivo

**Figure 4:**
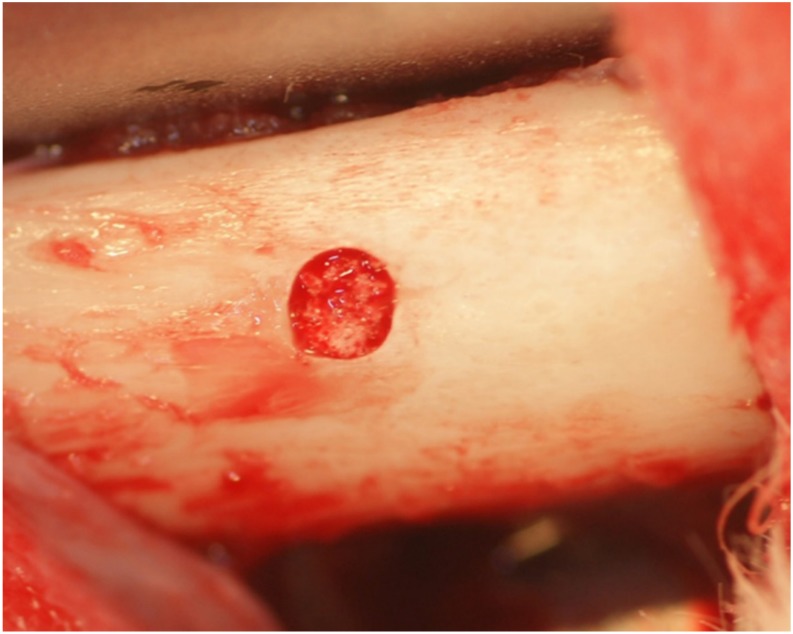
Sample placed into the bone socket of the rabbit femur.

Four male New Zealand white rabbits (3.0–3.5 kg) were used for the in vivo assessment of bone formation abilities of the developed materials. The animal study was approved by the Research Facilities Committee for Laboratory Animal Science, Hiroshima University School of Medicine (Approved number A11-5-5). The surgical procedures were performed on rabbits under general anesthesia with sodium pentobarbital (10 mg/kg) and local infiltration anesthesia with 2% lidocaine and 1:80,000 noradrenaline. Muscle and periosteal flaps were made on the left and right femurs, and a bone defect (diameter, 3 mm; length, 3 mm) was formed on both sides. The porous small-type phosphate treated HA and porous-HA (control) were respectively placed into the bone defects randomly (Figure 4).

**Figure 5:**
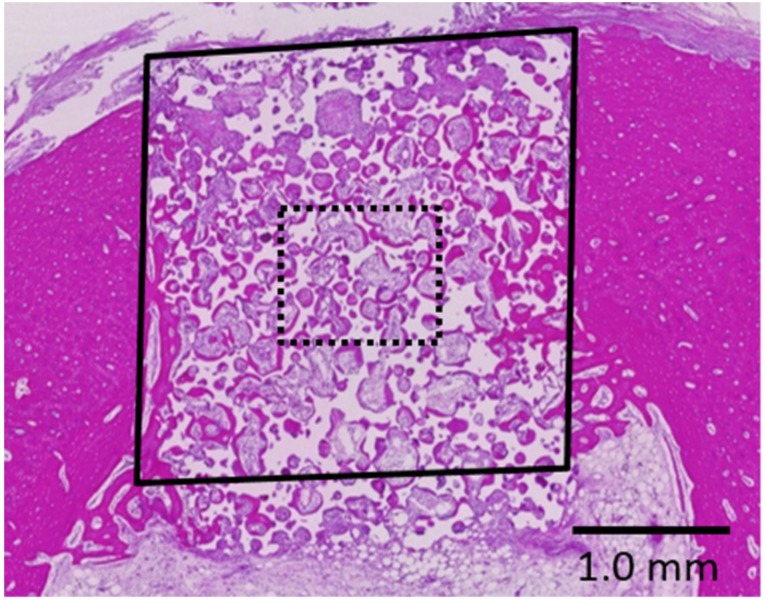
Schema of the histomorphometric analysis. The bone formation area was measured over the whole (solid-line square) and central (dotted-line square) area of the bone defect, calculated as the ratio of the area of newly formed bone to that of formed tissue area in the pores. The empty areas represent the scaffolds that had been removed by decalcification.

At 2 weeks after placement, the rabbits were anesthetized and perfused with 10% neutral formalin through the aorta. The femurs were harvested and further fixed in 10% neutral formalin for 1 week. Tissue blocks from each bone socket were cut and decalcified by treatment in K-CX solution (FALMA, Tokyo, Japan) for 1 week, dehydrated through a graded ethanol series, cleared with xylene, and embedded in paraffin. Sections with a 5-μm thickness were obtained from each block and stained with hematoxylin and eosin. Histological analysis was performed by light microscopy (BZ-9000, Keyence, Osaka, Japan). Images of bone regeneration were digitized and histomorphometrically analyzed using ImageJ software (National Institutes of Health, Bethesda, MD, USA). New bone formation in the pores of the scaffolds was quantified across the defect area and in the central area based only on the ratio of bone formation area to the total defect area (Figure 5).

### 4.4. Statistical Analyses

The differences in bone formation ratios were statistically compared by one-way analysis of variance and the Student *t*-test, evaluated at the 5% significance level.

## 5. Conclusions

Phosphate treated porous HA has a superior surface topography and bone formation abilities in vivo owing to the capacity for both osteoconduction and stimulation abilities of bone formation conferred by phosphoric acid etching.

## Figures and Tables

**Table 1:** Characteristic properties.

Group	The Porosity (%)	The Compressive Strength (MPa)
Porous HA	75.31 ± 1.62	6.98 ± 0.79 **
Phosphate treated HA	77.71 ± 4.68 *	2.72 ± 0.81

SD: standard deviation; * *p* = 0.0412, ** *p* = 0.0003.

**Table 2:** The bone formation ratio.

Group	Whole Area	Central Area
Porous HA	37.2 ± 4.5	37.4 ± 3.9
Phosphate treated HA	44.5 ± 7.7	47.8 ± 7.6 *

SD: standard deviation; * *p* = 0.0272.
